# Correction: McKay et al. Parent Education for Responding to and Supporting Youth with Suicidal Thoughts (PERSYST): An Evaluation of an Online Gatekeeper Training Program with Australian Parents. *Int. J. Environ. Res. Public Health* 2022, *19*, 5025

**DOI:** 10.3390/ijerph21010069

**Published:** 2024-01-08

**Authors:** Samuel McKay, Sadhbh J. Byrne, Alison Clarke, Michelle Lamblin, Maria Veresova, Jo Robinson

**Affiliations:** 1 Orygen, Parkville, VIC 3052, Australia; 2 Centre for Youth Mental Health, The University of Melbourne, Parkville, VIC 3010, Australia; 3 Centre for Global Health, Trinity College Dublin, D02 K104 Dublin, Ireland

There was an error in the original publication [1]. The Stigma of Suicide Scale short form (SOSS-SF) scale was scored incorrectly, as all 16 items of the scale were used. The correct calculation should have only included the eight items of the Stigma subscale. The significant changes in suicide stigma identified through linear mixed-effects modelling were no longer significant when the correct eight item subscale scale score was used. Corrections to the relevant sections are outlined below.

The authors state that the scientific conclusions are unaffected. This correction was approved by the Academic Editor. The original publication has also been updated.

## Text Correction

1. The was an error in the original publication. The abstract stated there were “reductions in suicide stigma”. However, no changes were identified in suicide stigma when using the corrected scale score. A correction has been made to the Abstract.

**Abstract:** The gatekeeper training of parents is a promising approach for suicide prevention in young people, but little research has addressed the effectiveness of such training, especially using online delivery. This study aimed to evaluate the efficacy and acceptability of the delivery of an online suicide prevention training program, LivingWorks Start, to improve the capacity of parents to support young people at risk of suicide. The participants were 127 parents of young people aged 12–25 who completed the LivingWorks Start training and consented to participate in the evaluation. The participants completed online surveys before, after, and 3 months after training. The participants showed increases in perceived self-efficacy and formal help-seeking intentions but no change in suicide stigma. Suicide literacy also increased, but only at the three-month follow-up. Most parents found the training acceptable, and did not find it upsetting. Prior mental health, suicide-related experiences, and pre-participation vulnerability were not predictive of finding the training distressing. Overall, the findings show that online gatekeeper training for parents can be beneficial, and is rarely associated with distress.

2. There was an error in the original publication. When describing scale scoring for the Stigma of Suicide Scale, it was stated that “The item scores were totaled to create a total suicide stigma score”, which was the incorrect scoring method for this scale, as only the eight items of the stigma subscale should have been used. A correction has been made to Section 2.4.5, paragraph 1.

Suicide stigma was assessed using the short form of the Stigma of Suicide Scale (SOSS) [20], which includes 16 items rated on a 5-point Likert scale ranging from one (Strongly disagree) to five (Strongly agree). In the current study, only the 8 items of stigma subscale were used, and the item scores were summed to create a total suicide stigma score. The possible range of the measure was 8–40, with higher scores indicating greater suicide stigma. Cronbach’s α in the present sample was 0.93.

3. There was an error in the original publication. When describing the results, it was stated that suicide stigma changed across time with the following: “In both the adjusted and unadjusted analyses (Table 1), self-efficacy, formal help-seeking, and suicide stigma all improved significantly from Time One to Time Two. However, only formal help-seeking did not decrease significantly from Time Two to Time Three, with self-efficacy and suicide stigma showing small but significant decreases.” However, when using the correct scoring method (e.g., summing the 8-item stigma subscale instead of summing all 16-items from the full scale), the observed changes in suicide stigma were no longer statistically significant. A correction has been made to Section 3.2, paragraph 1.

In both the adjusted and unadjusted analyses (Table 1), self-efficacy, formal help-seeking all improved significantly from Time One to Time Two. However, only formal help-seeking did not decrease significantly from Time Two to Time Three, with self-efficacy showing a small but significant decreases. Suicide literacy did not significantly increase from Time One to Time Two, but improved from Time Two to Time Three. Informal help-seeking and suicide stigma showed no change across time.

4. There was an error in the original publication. Table 1 showed significant changes in Suicide Stigma from Time One to Time Two and from Time Two to Time Three. However, when using the correct scoring method (e.g., summing the 8-item stigma subscale instead of summing all 16-items from the full scale), the observed changes in suicide stigma were no longer statistically significant. The corrected non-significant suicide stigma results are shown below. 

**Table 1:** Mean scores for outcome variables at each time point based on unadjusted and adjusted multilevel linear regression models.

	Unadjusted			Adjusted		
	Mean	95% CI ^a^	*p* Value ^b^	Mean	95% CI	*p* Value ^b^
**Self-Efficacy**						
Time One	60.12	56.85–66.39	-	60.67	57.52–63.83	-
Time Two	79.59	77.86–81.31	**<0.001**	79.89	78.19–81.59	**<0.001**
Time Three	77.58	75.78–79.59	**0.033**	77.86	75.79–79.94	**0.035**
**Formal Help-Seeking**						
Time One	17.99	17.43–18.55	-	18.03	17.46–18.60	-
Time Two	19.29	18.85–19.72	**<0.001**	19.32	18.89–19.76	**<0.001**
Time Three	19.03	18.53–19.54	0.325	19.03	18.53–19.53	0.254
**Informal Help-Seeking**						
Time One	16.75	15.71–17.78	-	16.87	15.87–17.87	-
Time Two	17.34	16.29–18.39	0.153	17.38	16.35–18.40	0.219
Time Three	17.79	16.74–18.85	0.415	17.79	16.76–18.81	0.467
**Suicide Stigma**						
Time One	9.92	9.15–10.68	-	9.84	9.07–10.61	-
Time Two	9.61	8.80–10.42	0.568	9.54	8.71–10.36	0.582
Time Three	9.63	8.87–10.39	0.958	9.54	8.78–10.31	0.998
**Suicide Literacy**						
Time One	0.86	0.84–0.88	-	0.86	0.84–0.88	-
Time Two	0.87	0.85–0.89	0.306	0.87	0.86–0.89	0.309
Time Three	0.90	0.88–0.91	**0.014**	0.90	0.88–0.91	**0.018**

^a^ Confidence interval; ^b^ tests the hypotheses that Time One scores differ from Time Two scores, and that Time Two scores differ from Time Three scores. *p* Values in bold represent significant effects at *p* < 0.05

5. There was an error in the original publication. It was stated that there was a significant change in suicide stigma based on the analyses conducted using the incorrectly scored scale. “Overall, the participants showed increases in perceived self-efficacy to prevent, or assist their child in managing, a suicidal crisis, formal help-seeking intentions for their child experiencing suicidal thoughts, and reduced suicide stigma. These changes followed the intervention (Time Two) and were maintained at the three-month follow up (Time Three), except for suicide stigma, which returned to the baseline at Time Three”. However, when using the correct scoring method (e.g., summing the 8-item stigma subscale instead of summing all 16-items from the full scale), the observed changes in suicide stigma were no longer statistically significant. A correction has been made to Section 4.1, paragraph 1.

This is the first study to assess an online suicide-specific education program, LivingWorks Start, with Australian parents. Overall, the participants showed increases in perceived self-efficacy to prevent, or assist their child in managing, a suicidal crisis, formal help-seeking intentions for their child experiencing suicidal thoughts, and reduced suicide stigma. These changes followed the intervention (Time Two) and were maintained at the three-month follow up (Time Three), except for suicide stigma, which returned to the baseline at Time Three. Suicide literacy also increased during the study, but this change occurred between Time Two and Time Three, and thus may not be related to the program. The participants reported no change in suicide stigma or informal help-seeking intentions for their child experiencing suicidal thoughts.

6. There was an error in the original publication. Based on the incorrect scale scoring and associated significant results it was stated that “This is noteworthy given that the current study found a small reduction in parents’ suicide stigma in the short-term, but this returned to baseline levels at the follow-up.” However, when using the correct scoring method (e.g., summing the 8-item stigma subscale instead of summing all 16-items from the full scale), the observed changes in suicide stigma were no longer statistically significant. A correction has been made to Section 4.2, paragraph 2.

Furthermore, although parents may feel more confident to intervene, it is important that their responses are perceived as appropriate by their children. Other Australian research [26] has found that parents’ responses to suicidal ideation disclosure were perceived as the least helpful of all informal/non-professional sources of support. This is noteworthy given that the current study found no change in parents’ suicide stigma. These findings suggest that training may need to further target stigma in order to ensure that parents do not intervene in a stigmatising or inappropriate manner, as this may increase the risk of their children perceiving their support as unhelpful. Taken together, the results suggest that it is crucial to examine both the impact of training on the actual behaviour of parents and how this behaviour is perceived by their children. While the present study did not have the opportunity to investigate actual behaviour, we recognise that this as an important direction for future research.

7. There was an error in the original publication. Based on the incorrect scale scoring and associated significant results it was stated that “First, the short-term follow-up period and absence of a control group means we cannot be sure that changes in self-efficacy, help-seeking intentions, suicide stigma or suicide literacy were retained long term or changed as a result of the training, as the effects could be a result of repeated testing.” However, when using the correct scoring method (e.g., summing the 8-item stigma subscale instead of summing all 16-items from the full scale), the observed changes in suicide stigma were no longer statistically significant. A correction has been made to Section 4.3, paragraph 1.

The findings of this study should be considered in light of several limitations. First, the short-term follow-up period and absence of a control group means we cannot be sure that changes in self-efficacy, help-seeking intentions or suicide literacy were retained long term or changed as a result of the training, as the effects could be a result of repeated testing. Second, the vast majority of the sample (89.9%) were female, which, while typical of this type of research [31], warrants caution when generalising these results to parents of all genders. Third, our sample may have been affected by the social media recruiting bias. For instance, samples recruited from social media have been noted to have an overrepresentation of Caucasian women [32], as well as higher education levels [33]. These characteristics were also highly prevalent in our current sample. It is also possible that self-selection bias played a role, with individuals who have lower levels of suicide literacy and higher levels of negative attitudes towards suicide being less likely to choose to participate. Fourth, there was only one Aboriginal and no Torres Strait islander participants, and only a small proportion of the sample was born outside of Australia, thus limiting the generalisability of the findings. Further research is needed to confirm the current findings and overcome the above-mentioned limitations by evaluating the LivingWorks Start program in a large-scale randomised control trial with more diverse samples.

8. There was an error in the original publication. Based on the incorrect scale scoring and associated significant results it was stated that “Such training may increase parents’ self-efficacy and help-seeking intentions for their children if they experience a suicidal crisis, while also reducing suicide stigma.” However, when using the correct scoring method (e.g., summing the 8-item stigma subscale instead of summing all 16-items from the full scale), the observed changes in suicide stigma were no longer statistically significant. A correction has been made to Section 5, paragraph 1.

This study provides preliminary evidence for the benefits of online gatekeeper training aimed at parents of young people. Such training may increase parents’ self-efficacy and help-seeking intentions for their children if they experience a suicidal crisis. Parents reported that the training was acceptable and most did not find it distressing. The online format appears to address barriers to research participation related to accessibility, but presents other challenges, such as privacy concerns. Future research using a randomised control design would facilitate stronger conclusions on the benefits of the program. Nevertheless, the current findings show that online suicide prevention training for parents is a promising avenue for future suicide prevention work.
